# English as a Foreign Language Teachers’ Identity and Motivation: The Role of Mindfulness

**DOI:** 10.3389/fpsyg.2022.940372

**Published:** 2022-06-28

**Authors:** Dianyong Zhu

**Affiliations:** School of Foreign Languages and Literature, Shandong University, Jinan, China

**Keywords:** EFL teachers, identity, mindfulness, motivation, education profession

## Abstract

Teaching is a career with a high rate of anxiety and burnout in all phases of teaching with specific challenges related to the feature of language education. The concept of motivation can be an important basic mechanism since educators who are not motivated are distressed because of the anxious characteristic of the education profession. Moreover, educator identity is a new issue that has built a perspective to examine educators’ growth by thinking about who they are as well as how they perform what they perform in a specific situation. Recently, programs that are based on mindfulness are known as efficient interventions enhancing motivation and decreasing tension in people. Given the prominence of mindfulness in educational settings and its neglected role in foreign language learning, the contemporary review attempts to inspect the function of mindfulness on English as a foreign language (EFL) teachers’ motivation and identity. Subsequently, some pedagogical recommendations for the educational representatives such as teachers and teacher trainers are offered.

## Introduction

Educators have important roles as the major actors in teaching and comprehending their opinions and principles is important since they face educational theories in the class (Derakhshan et al., 2020). The first stage for educators to be regarded as professionals is to determine their professional targets and wishes as Derakhshan et al. (2020) stated that educator identity is self-motivated which leads to a balance between their comprehending self in their career with their functions as educators. Edwards and Burns (2016) declared that educator identity is dynamic, multidimensional, and debated. In the same vein, as stated by Clarke (2009), educator identity is maintained to grow at the connection of the societal and the personal, discussion and practice, reification and involvement, similarity and contrast, agency and format, and offense, the individual and the numerous, and the synoptic and the dynamic. Moreover, the identity issue of foreign language educators is gradually becoming crucial because it is a significant key to guarantee the educators’ commitment to their job and compliance with the qualified norm (Hammerness et al., 2005). Being central to the education career, educator professional identity assists the EFL educators to form their own perspective regarding “how to perform,” “how to be,” and “how to comprehend” their profession and role in both school and community (Beauchamp and Thomas, 2009). Also, increasing a constructive professional identity assists learners to manage the demanding times of their careers. It is indicated that the concept of educator professional identity pertains to educators’ development to a large extent (Sheybani and Miri, 2019); therefore, some of the relevant issues affecting educators’ identities, such as educators’ motivation, engagement, incentive, adaptation, and satisfaction in their career should be taken into account (Danielewicz, 2001). As stated by Richardson and Watt (2018), motivation is a significant element in forming and developing an educator’s professional identity. Motivation is one of the main elements for educators, administrators, schools, and management to recognize all the exercises and writings stated before, which ultimately adds to learners’ success and innovation (Daniels, 2011; Davies et al., 2014; Asanjarani et al., 2021). Being conscious of the prominence of motivation in teaching and learning, Deci and Ryan (2016) have suggested self-determination theory (SDT) as a new perception and outlook for individuals’ motivation. Teacher motivation is an intricate idea regarded in the last decade and impacts student motivation and role (Dörnyei and Ushioda, 2011). A teacher with a decent level of motivation goes onboard and finishes his or her tasks with a sense of excitement and pleasure. The source of a decent level of motivation arises from within without requiring any interpersonal or intrapersonal enforcement (Neves de Jesus and Lens, 2005).

Consistent with some investigations, there is a strong connection between educator and learner motivation. In other words, if educators are greatly involved in their careers, learners are constructively impacted, and their education improves (Kalyar et al., 2018). Accordingly, enhancing educator motivation adds to bringing about the wanted educational setting in a class context. What results in educator motivation is not a unidimensional phenomenon (Budzińska and Majchrzak, 2021). Studies mention that economic conditions and level of income are of utmost significance to educators (Hildebrandt and Eom, 2011). In addition, motivation has an important role in building stronger identities among the educators since perceptible learning results and specific academic goals will be successfully achieved by the learners when the educators can have a greater level of enjoyment in their educational career (Deci and Ryan, 2016). Therefore, L2 educators’ motivation to teach must be improved and their mental requirements must be fulfilled so they can present well in their L2 classrooms. Indeed, previous studies (e.g., Claeys, 2011; Paulick et al., 2013) also highlight educator motivation as an impactful element in academic cycles.

To train more robust identities in teachers, and increase their motivation levels, they must accept any types of pressures, misfortunes, difficulties, and obstacles that could impede their growth (Ergas et al., 2018). The literature proposes that the strategies for learning teachers’ identity and motivation are different. For example, a large body of research has been carried out to ensure how teacher motivation can be improved to develop a better learning and educational context (Brown et al., 2002; Gordon, 2002). Mindfulness is one of the popular techniques that can help people control and regulate their tensions and adversities since it is acknowledged as mediation or treatment in positive psychology (PP) that has been applied in most domains and education (Pennock, 2017). A great amount of evidence has proved the advantageous influences of mindfulness on mental and physical well-being like enhancements in personal health and decreased anxiety and stress (Tomlinson et al., 2018). Mindfulness has attained increasing attention in academic psychology and teacher education (Ergas et al., 2018; Altan et al., 2019).

Mindfulness, the unprejudiced, present awareness of encounters, emerged as a flourishing management mechanism to decrease pressure and improve job and psychological health (Roeser et al., 2012; Rajabi and Ghezelsefloo, 2020). In the field of PP, even though there have been possible pros of mindfulness, little attention has been paid to educators’ mindfulness as one of the elements of educators’ health (Wang et al., 2021). Mindfulness in the specific setting of teacher education has been growing greatly lately, with most of its applications involving in-service educators and connected to the initial strand with expansions into societal-emotive capabilities (Jennings et al., 2017). In the last 20 years, the term mindfulness has taken place in the domain of teacher education with a developing body of studies on the cultivation of educator mindfulness through therapeutic interferences and meditation exercises (Lomas et al., 2017). As demonstrated by past research, educators’ mindful mental habits (e.g., pedagogical reflection in teaching, loving compassion toward learners, and acceptance of class transformation) seem to have a greatly constructive effect on their psychological health and prosperity, which can uphold the creation of constructive connections, enhance their continuous expert growth, and eventually can be advantageous to learners’ educational learning and individual development (Sharp and Jennings, 2016). For instance in some studies (Bluth et al., 2015; Jennings, 2015; Jennings and DeMauro, 2017), it is pinpointed that most educators struggle with motivation difficulties and mindfulness could be one of the factual mediations to make them conscious of themselves by mindful training. They argued that providing teachers with mindfulness training can help them cope better with stress at work and maintain a more productive classroom environment for learning. In this way, it is maintained that mindfulness can help teachers solve motivational issues in classrooms and mindful teachers will be more eager and willing to teach. Mindfulness can be encompassed by educators as a performance of teaching, where they attempt to build an association of meditation and directness with their learners and generate a classroom situation considered by significant relations between learners and the subject matter (Rodgers and Raider-Roth, 2006).

Although enough consideration is paid to the study of teachers’ identity and motivation among the factors influencing teachers’ careers, there is still a dearth of studies, particularly in the EFL context which considers the role of mindfulness. As a result, the current review examines if mindfulness helps educators have greater awareness regarding the present time and autonomously enhances their motivation as well as their identity.

## Review of the Literature

### Teacher Motivation

Motivation is regarded as a decisive factor affecting what students do and how well they perform it. An individual can be motivated by both external and internal motivations, where the latter leads to action or the fulfillment of specific conducts (Utvaer and Haugan, 2016). Motivation presents the main stimulant in language teaching and after some time the justification for looking for the extended and dull academic cycle (Dörnyei, 2005). Dörnyei (2005) added that motivation increases the whole elements related to gaining knowledge about a second language. In addition, it is stated that the motivation is associated with the reason behind peoples’ specific decision-making, engagement in a task, and persistence in following it and it manages the largeness of strong points and people’s engagement in learning a second language (Ushioda, 2008). To explain the performance of motivation in individuals, Ryan and Deci (2020) anticipated SDT in which the human conduct motivation is classified based on the internalization degree. Based on this theory, individuals control their behavior and personality development when experiencing different “kinds of motivation” (Ryan and Deci, 2020). Based on the hypothesis of self-determination, individuals with intrinsic motivation have an innate source of strength and inherent knowledge, are satisfied with succeeding in a task, are engaged in exercises leading to the encounter of stimulating emotions, and are willing to study new things (Deci and Ryan, 2016). Two different types of motivation exist, namely independent and regulated motivation. In one aspect, independent motivation alludes to action with a sense of decisiveness, facing selections and it is related to higher degrees of psychological well-being, higher will and decisiveness, higher mental skills, higher levels of professional consent, and organizational loyalty (Fokkens-Bruinsma et al., 2018).

High degrees of motivation will let the educators do more qualified educating-learning procedures where the learners can achieve the final objectives of their success (Salifu and Agbenyega, 2016). Moreover, two kinds of motivation are required to be constantly fostered to completely gain this major academic result, namely, internal and external motivation. Intrinsic motivation largely addresses the educators’ inner willingness to do their best in their present educating-learning activities. Therefore, the current educating-learning dynamics will enhance several important contributions for the educators to constantly endure in their education career. Educators’ education motivation can fall into two various types, namely, intrinsic and extrinsic motivation (Gultekin and Erkan, 2014). Thus, they state that specific conditions of educating-learning procedures experienced by the educators will enhance tangible effects for educators’ education motivation. But, intrinsic motivation cannot by itself sustain educators’ identities because external motivation can also lead to another vital effect for the educators to constantly revitalize their identities.

### Teachers’ Professional Identity

Identity pertains to how people describe themselves to themselves and others (Lasky, 2005). Identity is a dynamic concept with several aspects used to incorporate individual and others’ beliefs (Richardson and Watt, 2018). In addition, two kinds of identities exist, namely, individual and social identities. The former addresses how people describe their beliefs in some concrete measures, this is while the latter addresses how individuals incorporate their views and experiences to be tailored to specific societal conditions and societies’ prospects (Richardson and Watt, 2018). In an academic setting, the belief is that identity is attained obviously through educators’ societal settings and present academic settings (Richardson and Watt, 2018). Thus, specific characteristics obsessed by the educators are profoundly determined by important others following their life trips and all of these characteristics are constantly changing according to their current beliefs and measures. Such important change is attributed to attained identity because the educators are experiencing their measures and principles through important life skills. Educator identity is presumed to have an important function in judgments educators make regarding their teaching practices, the material they teach, and the type of connections they sustain with their learners (Izadinia, 2018). Identity is, therefore, regarded as an analytic lens through which to examine various dimensions of educators’ teaching presentation, the methods educators use to incorporate different effects piled up with time, the manners in which they solve pressure, and contrasts due to belonging to various societies and societal networks, accepting various principles, etc., and their probable manifestations in teaching (Beauchamp and Thomas, 2009). People’s reflection and social communication with others are vital to cultivate the educator’s professional identity, which in sequence turns into an instrument employed by educators to discuss and reflect on the societal dimensions of their roles (Aghaei et al., 2015). Some motivational scholars said that identity falls into two kinds, including ascribed and attained identity (Schutz and Hong, 2018).

There are three aspects of PI related to teachers’ disciplinary roles (van Veen et al., 2001). The first is educators’ awareness of their functions regarding scholastic purposes. Two particular orientations are theorized: a teacher direction with the primary emphasis on increasing learners’ subject information and abilities for qualification determinations and a teacher direction with a great emphasis on students’ well-being and general personal improvement (Popper-Giveon and Shayshon, 2017). The second one is teachers’ awareness of their functions concerning teaching; primarily directions toward teaching techniques (van Veen et al., 2001). Teachers might take a student/academic-centric direction where there is more emphasis on the student’s dynamic role in information development and in the academic method or a content/teacher-centric direction where more emphasis is on delivering a set group of information and abilities. The third aspect is educators’ awareness of their roles concerning the professional information basis. Teachers may view themselves as subject matter experts who take superiority in their robust foundation in subject information and skills, or view themselves as didactic and academic professionals who appreciate students’ academia the most and arrange their knowledge in generating purposeful and prominent subject academic experiences for learners (Lachner et al., 2016). Students’ PI is situated at the core of the academic profession since it offers teachers a system to develop their very own ideas regarding the way to be, the way to act, and the way to comprehend their job and their role in society. Importantly, teacher identity is not unchanging or forced; nevertheless, it can be debated through experience and the emotion that is created by such experiences (Sachs, 2005).

### Mindfulness

Mindfulness is identified in the field of PP and it has fundamental advantages such as enlightening working memory, stimulating well-being, and decreasing stress level (Brown and Ryan, 2003). Mindfulness refers to a situation of concentrating and understanding what is happening currently (Brown and Ryan, 2003) that has main features, such as target, presence, and acceptance, and the description of mindfulness is “continues consciousness,” a situation of bodily freedom, or continues consciousness of peoples’ unbiased experience (Katz, 2014). Mindfulness includes three vital components: intent, awareness, and attitude (Shapiro and White, 2014).

Mindfulness pertains to senses of strength and self-respect. People with high degrees of mindfulness are commonly expected to have a greater thinking-sensing adaptation and enhanced self-efficiency (Hosseinzadeh et al., 2019). It is held that instructing mindfulness begins with awareness or deliberately concentrating on something and that it is the foundation of mindfulness (Malinowski, 2013). It is the planned measure of focusing on momentary thoughts, affection, and measures (Kabat-Zinn, 2003). Intent refers to recognizing what and why something is conducted, or the final objective or perspective. Attention includes focusing entirely on the moment as is contrary to reflecting on the past events or what is going to happen. Attitude presents the ability to stay sympathetic, curious, and receptive. During mindful activities, all these three components are interconnected and intertwined. Promoting one’s immune practices, mental development, attention capabilities, and emotional control are the benefits of exercising mindfulness (Rechtschaffen, 2014).

Mindfulness is the deliberate focus of awareness, that refers to the present moment with an unbiased attitude to everything that happens in the present moment. Mindfulness is particularly common in the field of constructive psychology with vital benefits, such as improving active memory, enhancing health, lowering stress, etc. (Brown and Ryan, 2003). Mindfulness has been also efficient in bringing about healthy responses to nervousness and has decreased self-harm (Britton et al., 2014) which is an influential tactic for enhancing concentration and gratitude in educational spaces and permits teachers to use their time with higher efficiency. Mindfulness also assists learners to develop their mindfulness of the present time and it lays the groundwork for the mind to construct self-consciousness (Goleman, 2008).

## Conclusion

As stress directly affects teachers’ identity formation progressions (Pillen et al., 2013), developing tactics and overcoming such distress is vital for educators to familiarize themselves with the teaching career and its background. Altogether, this review emphasizes the importance of nurturing mindfulness as one of these techniques in learning and educating procedures. The importance of cultivating mindfulness in education and teaching cycles is emphasized in the literature. Certainly, a successful academic framework must ensure the growth of students’ self-satisfaction during education and offer learners more achievements; thus, mindfulness must be exercised in academic settings. Mindfulness is embedded in the theory of self-control (Brown, 2017). Elevating educators’ skill of recognizing their anxiety-provoked conduct and then changing their conduct toward their objective, could elevate educators’ degree of managing anxiety that could result in more motivation. Because mindfulness exercises provide teachers with possible abilities for dealing with anxiety, they can be utilized as pedagogical instruments in the class to increase learners’ health and motivation offering all pre-service and in-service educators the chance to train for mindfulness easily when it comes to their career. Therefore, it can be inferred that mindfulness exercises are effective ways to coach educators to advance flexibility, lessen anxiety and motivation, and associate with their learners and themselves. Jennings (2015) maintained that, by employing mindfulness in their work with an open-hearted, current-moment, indulgent consciousness, all together teachers can make a big variance in their classroom settings and their students’ achievement.

Enhancing awareness and attention of the ideas, feelings, and trends are among some of the positive dimensions of mindfulness, leading to the organization of the coping behaviors and the constructive emotional moods; it even enhances the people’s capability to involve in personal and social practices and their awareness in these tasks. This training, thus, can alter individuals’ attitudes toward incidents and actions by enhancing the mentioned capabilities and inspiring them to take part in personal and social tasks and be influential in enhancing their career presentation (Sharf, 2009). A large body of study and discussions regarding mindfulness in educators concentrates on decreasing the level of tension. However, the pervasive possibility of mindfulness exceeds tension relief to strengthen the educators to specify and react to the basic causes of tension. Mindfulness assists specify when the source of such tension is the cognitive dissonance which is caused by the separation between individual internal values and external vocational values. In addition, it assists to solve such problems by providing a more complete and transparent comprehension of the outline of its terrain as the current experience of that dissonance in one’s body, heart, and mind are investigated. Instead, such depth of comprehension assists the practitioner to make decisions that resolve intellectual dissonance by letting deeper incorporation of individual internal and vocational external values, instead of merely preferring vocational values over individual ones, therefore, letting the advent of a more authentic vocational identity which is well-grounded as well as persistence through time.

Scholars have ultimately begun an empirical study on the possible advantages of mindfulness for the education career and their study has tracked the wider empirical literature on mindfulness by focusing on tension, concentration, and wellbeing. But, the theory of cognitive dissonance and its implications for educators indicates that mindfulness may also have the ability to strengthen educators to specify and resolve the pressure and anxiety between their individual internal principles and the external reward-based values in their career such that it permits for the advent of their authentic vocational identity.

Promoting and boosting mindfulness has a special function in identity growth, promoting an experimental self, which offers a novel viewpoint in life and allows for novel insights about a person’s narrative self. Mindfulness coaching can be regarded as a combinative pedagogy for genuine individual and expert identity growth in educator training. Even though mindfulness originates from Buddhist psychology, the exercise of paying attention is innately a secular practice that is studied positively. Studying mindfulness can be viewed as an important cognizance that helps educator candidates with enacting societal justice and equity in their class.

## Implications and Suggestions for Further Research

Mindfulness instruction can be highly beneficial to EFL teachers since mindless motivates them, and renders them more goal-directed to achieve in their professions. Mindfulness overall decreases stress and concern and assists with reducing language-relevant anxiety. This has thus been shown to result in improving EFL teachers and consequently learners’ performance. In addition, People with great degrees of mindfulness can also promote motivation (Ruffault et al., 2016). Mindfulness assists educators with lessening anxiety, enhancing their energy levels, lessening their deconstructive thoughts, and building a more constructive life demeanor that elevates their motivation and recognition of the current. Educators who had mindfulness attributes also had a more exceptional skill to encounter their issues, carry out their obligations, conquer impediments and stay away from using excuses that could keep them from attaining their objectives. EFL educators who have greater degrees of mindfulness have less tension, anxiety, and depression; their degrees of burnout is decreased which is a significant drawback in education careers. They have a better involvement with their learners and they understand to get curious and attracted to their activities instead of only cramming their brains with data. Mindful educators learn to concentrate their awareness on what they desire. Simultaneously, they enjoy higher productivity. It is maintained that teachers with greater mindfulness can notice, change, and manage their intellectual and affective experiences in a way that leads to less pressure, sadness, and stress. It is theorized that less anxiety results in newfound strength that teachers can use to promote positive connections with students. The outcomes of this study uphold these theories, and so, support the present work that aims at teachers’ mindfulness as a resilient factor in professional advancement plans for lessening teacher pressure and improving teacher-student associations inside the classroom (Roeser et al., 2012). With methods of mindfulness education, educators can find happiness and love in education and cooperate with learners due to their better perspectives and a higher level of motivation. Mindfulness methods are beneficial for educators to soothe their minds, relate more with learners, and regain passion and happiness in education and interaction with learners.

Teacher trainers should try to include mindfulness in the professional development programs since mindfulness in education can be a segment of educator training programs of the educators, and such ability can also be nurtured in educators employed in the field. Also, one application exists for academic policy-makers and they have to accept the perspective that mindfulness intervention acts as a contributing instrument for educators for whom efficient education and their learners’ effective education are highly important. Therefore, academic decision-makers and officials have to admit the mindfulness intervention as a way to efficacy and they should integrate mindfulness-improving tactics into the class and instruct educators in integrating these techniques within class activities. Moreover, institutes looking for various methods to help their educators in promoting their standard of motivation can also benefit from this research. They can apply similar mindfulness interference plans to enhance their educators’ motivation. Motivated educators are well involved in their careers and devoted to their learners’ education. Implementing mindfulness and attaining abilities that add to anxiety control, flexibility, and career fulfillment is the precursor to this problem and develops the culture of increasing and sustaining motivated, quality educators (Skaalvik and Skaalvik, 2016).

Future scholars need to investigate the associations between constructive features in educators, pertaining variables, and mental capital thru various measurement instruments to cultivate a better comprehension of constructive features in educators and their association with motivation, identity, and mindfulness. Even though this review scrutinized the effect of mindfulness on teacher identity and motivation in teaching, research on the effect of learners’ emotional and social procedures will be also significant for the future. Hopefully, research on the efficiency of teaching programs to improve mindfulness in education can assist the social mindfulness in literature. Additionally, mixed-method research could be beneficial in attaining qualitative data involving educators’ recognition of health, exhaustion, and mindfulness in addition to other suggestions for educators. The qualitative dimension of mixed-method research could also be advantageous in getting feedback on the mindfulness coaching plan and validating the quantitative outcomes of the present research. Utilizing interviews can elucidate our comprehension to determine what potential mental factors can be impacted in creating and building educators’ expert identity, regarding their motivation and directions.

## Author Contributions

The author confirms being the sole contributor of this work and has approved it for publication.

## Conflict of Interest

The author declares that the research was conducted in the absence of any commercial or financial relationships that could be construed as a potential conflict of interest.

## Publisher’s Note

All claims expressed in this article are solely those of the authors and do not necessarily represent those of their affiliated organizations, or those of the publisher, the editors and the reviewers. Any product that may be evaluated in this article, or claim that may be made by its manufacturer, is not guaranteed or endorsed by the publisher.
